# Mapping the Level of Evidence of Prenatal, Childhood, and Adolescent Exposure to Volatile Organic Compounds and Health Outcomes: Protocol for a Scoping Review

**DOI:** 10.2196/71587

**Published:** 2025-06-06

**Authors:** Homègnon Antonin Ferréol Bah, Emily Leydet, Mackenzie L Connell, Andrea E Cassidy-Bushrow, Jennifer K Straughen

**Affiliations:** 1 Department of Public Health Sciences Henry Ford Health Detroit, MI United States; 2 Department of Environmental & Global Health College of Public Health and Health Professions University of Florida Gainesville, FL United States; 3 Department of Pediatrics and Human Development College of Human Medicine Michigan State University East Lansing, MI United States; 4 Henry Ford Health + Michigan State University Health Sciences Detroit, MI United States; 5 Department of Obstetrics, Gynecology and Reproductive Biology College of Human Medicine Michigan State University East Lansing, MI United States

**Keywords:** benzene, BTEX, child health, environmental health, ethylbenzene, human (0 to 18 years), PCE, pregnancy outcomes, toluene, trichloroethylene, tetrachloroethylene, perchloroethylene, VOCs, TVOCs, xylene

## Abstract

**Background:**

Volatile organic compounds (VOCs) are a diverse group of organic chemicals with widespread presence in daily life. VOCs may have detrimental health effects on humans, particularly during critical periods such as pregnancy, childhood, and adolescence. Although the body of evidence is growing, significant gaps remain in the current understanding of the associations of VOCs on health across these developmental stages. Moreover, existing reviews focused on specific VOCs or individual health conditions, highlighting the need for a comprehensive summary of the current literature to stimulate further research.

**Objective:**

This ongoing scoping review aims to map the current evidence concerning exposure to VOCs and their associations with pregnancy outcomes and health during pregnancy, childhood, and adolescence.

**Methods:**

This review will consider studies investigating potential health effects related to exposures to either total VOCs or any of the following 6 specific VOCs: benzene, toluene, ethylbenzene, xylene, trichloroethylene, and tetrachloroethylene or perchloroethylene during critical windows from pregnancy through adolescence. Original articles published in English from January 1, 2000, through the end of the scoping review period, will be included. We will follow the Joanna Briggs Institute methodology for scoping reviews and the Preferred Reporting Items for Systematic Reviews and Meta-Analyses extension for Scoping Reviews (PRISMA-ScR) guidelines. The literature search will use the Ovid MEDLINE, Embase, and Web of Science databases to identify relevant citations. The following steps of the review will be managed through the Covidence platform. A total of 2 reviewers will independently conduct a rapid screening of paper titles, before evaluating the abstracts and full texts to determine which articles will proceed to the data extraction stage. In case of discrepancies during these steps, the reviewers will discuss to reach a consensus, and a third reviewer may intervene if needed. Reviewers will provide commentaries on their findings from the included studies and suggest areas of interest for future studies.

**Results:**

The literature search yielded a total of 7332 citations published during the period January 1, 2000, to July 25, 2024. After the title screening and reading the abstract and full text, 274 papers were selected for the data extraction phase, which is currently underway. Upon completion of the library’s updated literature search, covering July 26, 2024, to the present, we will proceed with data analysis and manuscript writing for submission to a peer-reviewed journal.

**Conclusions:**

The results are expected to summarize the current state of knowledge on the association of VOCs with health at critical life stages (pregnancy, childhood, and adolescence). We anticipate identifying gaps in the literature as well that could guide future research priorities.

**Trial Registration:**

OSF Registries 10.17605/OSF.IO/EP73G; https://osf.io/ep73g

**International Registered Report Identifier (IRRID):**

DERR1-10.2196/71587

## Introduction

Volatile organic compounds (VOCs) represent a broad, diverse group of organic chemicals characterized by their ability to vaporize at room temperature [1,2]. While some VOCs occur naturally, most human exposure is through anthropogenic activities and their presence in personal care products. VOCs arise from culinary activities, construction materials, furniture, cleaning products, cosmetics, fuels, and treated wood [3-5]. Humans are exposed through respiratory, dermal, and oral routes [3]. VOCs such as benzene, trichloroethylene, ethylbenzene, toluene, and xylene have been classified as carcinogens or mutagens [1] and are associated with a wide range of health effects, including damage to the neurological, respiratory, and immunological systems [1,4]. Most of the knowledge on the effects of VOCs on human health is from studies focused on occupationally exposed individuals [1,6,7]. The widespread presence of VOCs in daily life has raised concerns about the potential health effects on the general population, particularly during pregnancy and in critical developmental periods [8-10], when rapid growth and development coupled with immature physiological systems may render individuals more susceptible to adverse exposures. This may lead to deleterious consequences even later in adulthood, such as cardiovascular and other chronic diseases (eg, obesity and diabetes) [11].

A rapid preview search of the literature for articles published between 1990 and 2024 (June 5) (via PubMed) on the health effects of VOC exposure on pregnant females and children showed that most research was conducted during the last 3 decades, with only 1 study published in 1995 [12]. There is a need to monitor the current level of evidence on the impact of VOCs on human health during critical life stages to provide accurate information and guide future research. Most available literature reviews on VOCs focused on a few specific VOCs, diseases, or demographic groups [13-18]. We suggest here a scoping review with no restriction on prespecified health effects associated with the following 6 VOCs prioritized by the Center for Leadership in Environmental Awareness and Research (CLEAR) [3] funded by the Superfund Research Program of the National Institute of Environmental Health Sciences (NIEHS): benzene, toluene, ethylbenzene, xylene, trichloroethylene, and tetrachloroethylene/perchloroethylene [19]. Studies with total VOC measurements will be also included. This review will include exposures and health effects that occur to pregnant females or children from conception through adolescence. Searches of the PROSPERO and Cochrane databases showed no ongoing systematic or scoping review registered with similar topics or goals. We aim to (1) describe the current level of evidence regarding the health effects of exposure to benzene, toluene, ethylbenzene, xylene, trichloroethylene, tetrachloroethylene or total VOCs in humans during pregnancy and from conception to adolescence; and (2) identify key knowledge gaps to propose priorities for future research studies on the health effects of VOC exposure during critical developmental periods.

## Methods

### Overview

This review will follow the Joanna Briggs Institute methodology for scoping reviews and the Preferred Reporting Items for Systematic Reviews and Meta-Analyses extension for Scoping Reviews (PRISMA-ScR) guidelines [20,21].

### Participants

We will consider studies conducted on associations between exposure to the 6 VOCs of interest (benzene, toluene, ethylbenzene, xylene, trichloroethylene, and tetrachloroethylene) and/or total VOCs and health effects in pregnant females and on the child from conception through 18 years. Table 1 presents the Population, Exposure, and Outcomes (PEO) framework for this review.

**Table 1:** Population, Exposure, and Outcomes (PEO) framework.

Elements	Definition
Population	Pregnant females, fetuses, and individuals ages 0 to 18 years (ie, infant, child, and adolescent)
Exposures	Benzene, toluene, ethylbenzene, xylene, trichloroethylene, and tetrachloroethylene and derived products and total VOCs
Outcomes	Any health outcome, including birth outcomes

### Concept

Regardless of the assessment method—whether derived from questionnaires, screenings, biomarkers (including epigenetic markers such as DNA methylation changes), laboratory tests, or clinical diagnoses—any health outcome will be considered for this review. Only the relationship of the VOCs with any health effect will be reported. Studies with total VOC measurement will be included, as this method of exposure assessment represents a proxy for the pollutants considered in this review. These will be reported as their own exposure category. Studies using assessment of exposure to VOCs through biological, environmental matrices, or mathematical or geospatial modeling (eg, exposure estimation based on residential address) will be considered. Exposure and health effects occurring during and throughout adulthood (in the nongravid state) will not be considered.

### Context

Studies done in any part of the world and published in English will be included. Both occupational and nonoccupational exposures will be examined.

### Types of Sources

This scoping review will consider studies that include primary and secondary data, with only peer-reviewed original articles included. All epidemiological research designs such as cross-sectional, retrospective, prospective, bidirectional cohort studies, and case-control studies, and their variations will be explored. Systematic reviews, meta-analyses, and gray literature will not be included in this review (Textbox 1).

**Inclusion and exclusion criteria.**

**Inclusion criteria**

- Peer-reviewed original articles.
- Studies conducted in humans at specific life stages described by the Population, Exposure, and Outcomes (PEO) framework (Table 1).
- Studies assessing exposure to volatile organic compounds (VOCs) through biological, environmental matrices or mathematical or geospatial modeling will be considered.
- Papers published from 2000 to the date of the end of the extraction process (likely first quarter of 2025).

**Exclusion criteria**

- Articles not published in English.
- Papers that are not empirical studies (eg, review articles, conference abstracts, editorials, and commentaries).
- Studies conducted in animals or adult (nonpregnant) humans (>18 years).
- Studies assessing or estimating exposure to VOCs solely through questionnaires; occupation history (ie, no direct estimate of the 6 specific or total VOCs).
- Studies that use risk assessments (for cancer or noncancer outcomes) based on indices such as the target margin of exposure or hazard quotient as their primary measure of health outcomes.

### Search Strategy

The literature search will be conducted to find original research articles with primary or secondary data. A previous rapid literature search with the support of the Sladen Library has been conducted through PubMed MEDLINE to identify reviews (systematic, scoping, and meta-analysis) and original papers to define adequate strategies for the scoping review process. The keywords used are presented in Multimedia Appendix 1. The presearch yielded 265 papers from 1990 to 2024 (June 5), from which 103 papers were eligible for full-text reading and data extraction. As only 1 manuscript (published in 1995) was eligible for the period from 1990 to 2000, we will focus on the period from 2000 to the present for this proposed review.

Literature for the proposed review will be identified by searching Ovid MEDLINE, Embase, and Web of Science. Our literature search will follow the strategy described by Liu et al [22] in their systematic review of indoor VOC exposures and health impacts. The Covidence platform (Covidence systematic review software, Veritas Health Innovation, Melbourne, Victoria, Australia) [23] will be used to screen references, extract data, and track the progress of the work.

### Study Selection

After the initial search conducted by the Sladen Library, the citations will be uploaded on the Covidence platform. A total of 2 reviewers will independently conduct a rapid screening based first on the paper’s titles, removing those that are not relevant or do not meet inclusion criteria. Discrepancies will be determined by a third reviewer. Next, 2 reviewers will evaluate the articles’ abstracts and full text for relevance. Each article will be reviewed by 2 reviewers during the full-text review stage. The reviewers will document reasons for the exclusion of any full-text article. Any discrepancies in exclusion or inclusion decisions will be discussed by 2 reviewers and, if necessary, adjudicated by a third reviewer. At the end of this process, the reviewers will examine the bibliographies of the relevant papers (selected for the extraction process) to identify additional articles pertinent to the study objectives. Citations will be managed using EndNote 21 (Clarivate).

Full-text articles must be available to be included in the final analysis of this review. If any publication is unavailable through the Sladen Library resources, we will contact the publication’s corresponding author to request the paper’s full text. For transparency, all details on the results of contacting authors of the unavailable papers will be provided in the methodology section of the final version of the publication resulting from this research. After the data extraction and synthesis, the Sladen Library will conduct a supplementary search to consider any possible new evidence.

### Data Extraction

The remaining articles will undergo full-text review for further data extraction and synthesis. To define the level of evidence in this scoping review, the following information will be collected as shown in Textbox 2: metadata (authors, country, and year of publication), aims of the study, list of VOCs investigated, methodology (research design, sample size, measures of association, methods to assess VOC exposure and health outcomes, potential confounders), and main conclusion.

A total of 2 reviewers will extract the information separately. In case of discrepancy, both will discuss to reach a consensus. If needed, a third reviewer may intervene. This data extraction instrument may be modified if required according to its efficiency.

**Draft data extraction instrument.**

**Information to be extracted**

- Authors, country, and year of publication
- Setting (Occupational vs nonoccupational)
- Aims or purpose
- Volatile organic compounds (VOCs) investigated (and VOC mixtures, if applicable)
- Health outcomes investigated
- Participants (age) and sample size
- Study design
- Method of assessment: exposure and outcome
- Covariables or confounders considered
- Main conclusion or findings

### Data Analysis and Presentation

This section will address the 2 main review questions. Considering the level of evidence, results will be shown as descriptive data (absolute and relative frequency) of the characteristics of the articles extracted, such as the following:

1. Study design: cohort, case-control, cross-sectional, and others.
2. Techniques used to assess:
3. Exposure: biomarkers (eg, VOC metabolites in blood, urine) or environmental matrices (eg, VOC levels in air, water); ie, direct or estimated exposure.
4. Health outcome: disease presence or absence, severity, overall health or specific function, biomarkers of disease or health.
5. Measures and strength of association: such as relative risk, odds ratios, attributable risk, CIs, and *P* values.

The goal is to describe the current rigor and depth of previous investigations. We will use a table, chart, or figure to highlight our main findings.

To highlight the current gaps and perspectives for future studies, our results will be presented as a conceptual and thematic map considering the 3 components of our PEO framework (Table 1). The overall goal of the map is to highlight aspects of the associations between VOCs and health in critical windows that have been studied in detail as well as those lacking attention.

We will present a narrative review of the literature describing the similarities and differences in studies conducted on the same topic to highlight possible reasons for contradictory or inconsistent findings. Considering the growing interest in studying exposure to pollutant mixtures, we will present data on the number of studies that evaluated exposure mixtures and those that report mixtures that include at least one of the 6 VOCs of interest, and we will summarize both the associations with health outcomes and the methods used to evaluate mixture effects. Finally, we will comment on and suggest possible implications of this scoping review for future research.

## Results

To date, a comprehensive literature search, a title screening and a full-text review have been conducted, yielding respectively 7332 citations, 538 full texts, and 274 papers to be extracted. This protocol has been registered on the Open Science Framework [24]. Data extraction is expected to be completed by July 2025. A complementary literature search will be conducted by the library, covering July 26, 2024, to the present, followed by data analysis and preparation of the final report. We anticipate submitting the results for peer review by September 2025 and plan to disseminate the findings through conference presentations and other scholarly platforms.

## Discussion

### Anticipated Findings

We present the protocol for an ongoing scoping review aimed at evaluating health outcomes associated with 6 VOCs (benzene, toluene, ethylbenzene, xylene, trichloroethylene, and tetrachloroethylene) and total VOCs, which are priorities of an NIEHS-funded Superfund Research Center, CLEAR [19]. This review specifically focuses on pregnant females, fetuses, and individuals ages 0 to 18 years and seeks to address key gaps in knowledge.

To our knowledge, no prior review has provided a comprehensive summary focusing on these VOCs during specific critical life stages. While Liu et al (2022) conducted an extensive systematic review of 8 VOCs, their focus was restricted to indoor exposures and health outcomes at all life stages [22]. Other reviews (systematic, meta-analyses, or narrative) have considered VOCs as part of broader air pollution studies [25-28] or focused narrowly on specific aspects such as (1) individual VOC exposures [6,29-31], (2) specific organ systems [18,31], (3) specific health outcomes such as cancer, respiratory diseases, or allergies [13,26,28,32], or (4) specific populations or geographic regions [16]. In contrast, our review will provide a broader and more inclusive analysis of VOC-related health outcomes in critical stages of human development. Emerging paradigms, such as the life-course perspective [33] and the Developmental Origins of Health and Disease (DOHaD) framework [34], underscore the importance of early prevention. These approaches aim to minimize environmental exposures during key developmental periods, reducing the risk of chronic diseases later in life. Focusing this scoping review on exposures and health outcomes from pregnancy through adolescence will guide future research in this area that could lead to primary prevention efforts.

This review will identify gaps in the current literature and facilitate the development of new research questions. For instance, it may reveal a lack of studies addressing the combined effects of VOCs and other environmental stressors, including chemical and nonchemical factors such as social determinants of health. By highlighting the current evidence and knowledge gaps, we aim to contribute to the dissemination of accurate information, address the limitations of current research, and support efforts to reduce health disparities.

This work has several limitations. First, there is a potential risk of bias, as no formal quality assessment will be conducted, given the nature of scoping reviews. In addition, restricting the language criteria to English limits the inclusion of literature predominantly to Western or English-speaking regions. This approach may exclude valuable evidence from non–English-speaking regions, such as countries in West and Central Africa, Southeast and Central Asia, and Latin America.

### Conclusion

This scoping review aims to synthesize evidence from studies conducted since the beginning of the 21st century examining associations of VOC exposures with health outcomes in pregnancy through adolescence. This review is expected to inspire additional research, particularly in these population groups, potentially accelerating the development of policies that promote health equity and protect vulnerable populations.

## Appendix

Multimedia Appendix 1 Search strategy for the pre-review process.

Multimedia Appendix 2 ChatGPT prompt samples for editing/revising manuscript language.

## Abbreviations

CLEAR: Center for Leadership in Environmental Awareness and Research
DOHaD: Developmental Origins of Health and Disease
NIEHS: National Institute of Environmental Health Sciences
PEO: Population, Exposure, and Outcomes
PRISMA-ScR: Preferred Reporting Items for Systematic Reviews and Meta-Analyses extension for Scoping Reviews
VOC: volatile organic compound
