# Supplementary material for: Mapping the Level of Evidence of Prenatal, Childhood, and Adolescent Exposure to Volatile Organic Compounds and Health Outcomes: Protocol for a Scoping Review
Source: JMIR Res Protoc. 2025 Jun 6;14:e71587. doi: 10.2196/71587 (PMC12181746; doi:10.2196/71587)
Supplement: Multimedia Appendix 1 [file resprot_v14i1e71587_app1.docx]

**Appendix I: Search strategy for the pre-review process**

Database:

Ovid MEDLINE(R)ALL<1946 to June 05, 2024> (only publication in English)

| # | **Query** | **Results**  **from 6 June 2024** |
| --- | --- | --- |
| 1 | *Volatile Organic Compounds/ae, an, po, to [Adverse Effects, Analysis, Poisoning, Toxicity] | 4,075 |
| 2 | adolescent/ or birth cohort/ or child/ or exp child, preschool/ or exp infant/ or pregnant women/ | 4,066,332 |
| 3 | environmental exposure/ or maternal exposure/ | 94,642 |
| 4 | Air Pollutants/ae, an, po, to [Adverse Effects, Analysis, Poisoning, Toxicity] | 51,654 |
| 5 | (infant* or child or birth or children or "early Life” or utero or pregnancy or maternal or prenatal or school* or pediatric or pediatric or preschool*).mp. | 4,620,845 |
| 6 | (benzene or toluene or ethylbenzene or BTEX or Trichloroethylene or Tetrachloroethylene).mp. | 83,191 |
| 7 | Benzene/ae, an, po, to [Adverse Effects, Analysis, Poisoning, Toxicity) | 4,550 |
| 8 | Toluene/ae, an, po, to [Adverse Effects,  Analysis, Poisoning, Toxicity) | 2,911 |
| 9 | Xylenes/ae, an, po, to [Adverse Effects, Analysis, Poisoning, Toxicity) | 1,448 |
| 10 | Trichloroethylene/ae, an, po, to [Adverse Effects, Analysis, Poisoning, Toxicity] | 2,081 |
| 11 | Tetrachlorethylene/ae, an, po, to [Adverse Effects, Analysis, Poisoning, Toxicity] | 812 |
| 12 | 6 or 7 or 8 or 10 or 11 | 83,769 |
| 13 | 2 or 5 | 5,660,279 |
| 14 | 3 and 12 and 13 | 481 |
| 15 | 1990 to June 05, 2024 | 265 |
