# Supplementary material for: Mapping the Level of Evidence of Prenatal, Childhood, and Adolescent Exposure to Volatile Organic Compounds and Health Outcomes: Protocol for a Scoping Review
Source: JMIR Res Protoc. 2025 Jun 6;14:e71587. doi: 10.2196/71587 (PMC12181746; doi:10.2196/71587)
Supplement: Multimedia Appendix 2 [file resprot_v14i1e71587_app2.docx]

**ChatGTP prompt samples for editing/revising manuscript language**

**You said:**

correct : This is a scoping review to map the state of art regarding the level of evidence concerning exposure to Volatile Organic Compounds during pregnancy, childhood and adolescence. This will be helpful to orientate the future research on the field as more awareness has been raised about health issues impacts of this broad group of contaminants.

**ChatGPT said:**

This scoping review aims to map the current state of the art regarding the level of evidence concerning exposure to volatile organic compounds during pregnancy, childhood, and adolescence. It will help guide future research in this field as awareness grows about the health impacts of this broad group of contaminants.

**You said:**

correct this text: As only one manuscript (published in 1995) was eligible in the period 1990 to 2000 and considering our frame time availability for this project completion we will consider for this review the period 2000- present.

**ChatGPT said:**

Here's the corrected text:

As only one manuscript (published in 1995) was eligible for the period from 1990 to 2000, and considering our timeframe availability for this project's completion, we will focus on the period from 2000 to the present for this review.

**You said:**

As only one manuscript (published in 1995) was eligible for the period from 1990 to 2000, and considering our 6 months timeframe availability for this project's completion, we will focus on the period from 2000 to the present for this review.

**ChatGPT said:**

Here's the revised text:

As only one manuscript (published in 1995) was eligible for the period from 1990 to 2000, and considering our six-month timeframe for this project's completion, we will focus on the period from 2000 to the present for this review.

**You said:**

The literature search will be conducted to find original research article conducted with primary and or secondary data. A previous rapid literature search with the support of the Sladen Library of Henry Ford Health has been conducted through PubMed MEDLINE to identified reviews (systematic, scoping, and meta-analysis) and original papers to define adequate strategies for the scoping review process itself. The keywords used are presented in the Appendix 1. The search yielded in 265 papers from 1990 to 2024 (June 05), from which 103 originals papers were eligible for full text reading and data extraction phase. As only one manuscript (published in 1995) was eligible for the period from 1990 to 2000, and considering our six-month timeframe for this project's completion, we will focus on the period from 2000 to the present for this review.

**ChatGPT said:**

Here's the corrected and revised text:

The literature search will be conducted to find original research articles using primary and/or secondary data. A previous rapid literature search, supported by the Sladen Library of Henry Ford Health, was conducted through PubMed MEDLINE to identify reviews (systematic, scoping, and meta-analysis) and original papers to define adequate strategies for the scoping review process itself. The keywords used are presented in Appendix 1. The search yielded 265 papers from 1990 to June 5, 2024, from which 103 original papers were eligible for the full-text reading and data extraction phase. As only one manuscript (published in 1995) was eligible for the period from 1990 to 2000, and considering our six-month timeframe for this project's completion, we will focus on the period from 2000 to the present for this review.

**You said:**

improve the text: • Articles not published in English • Paper which are not from empirical studies such as review papers, conference abstract, editorial and commentary paper • Studies conducted in animals or adult human (>18 years) • Studies using questionnaire to assess exposure to VOCs (i.e. none of the six specific VOCs is assessed). • Studies which conducted risk assessments as health outcomes (cancer or non-cancer diseases) through measurement of index such as target margin of exposure (MOE) and hazard quotient (HQ).

**ChatGPT said:**

Here is an improved version of the text: - Articles not published in English. - Papers that are not empirical studies, such as review articles, conference abstracts, editorials, and commentaries. - Studies conducted on animals or adults over 18 years of age. - Studies that assess exposure to VOCs using questionnaires (i.e., none of the six specific VOCs are measured). - Studies that evaluate health outcomes (cancer or non-cancer diseases) by using risk assessments based on indices such as the Target Margin of Exposure (MOE) and Hazard Quotient (HQ). for a few seconds

**Suggested Improvement:**

- Articles not published in English
- Papers that are not empirical studies (e.g., review articles, conference abstracts, editorials, commentaries)
- Studies conducted in animals or in adult humans (>18 years)
- Studies assessing exposure to VOCs solely through questionnaires (i.e., no direct measurement of the six specific VOCs)
- Studies that use risk assessments (for cancer or non-cancer outcomes) based on indices such as the target margin of exposure (MOE) or hazard quotient (HQ) as their primary measure of health outcomes
